# Lymphatic Endothelial Cell Activation and Dendritic Cell Transmigration Is Modified by Genetic Deletion of Clever-1

**DOI:** 10.3389/fimmu.2021.602122

**Published:** 2021-03-04

**Authors:** Sina Tadayon, Johannes Dunkel, Akira Takeda, Dominik Eichin, Reetta Virtakoivu, Kati Elima, Sirpa Jalkanen, Maija Hollmén

**Affiliations:** ^1^ MediCity Research Laboratory, University of Turku, Turku, Finland; ^2^ Turku Graduate School of Molecular Medicine, University of Turku, Turku, Finland; ^3^ Institute of Biomedicine, University of Turku, Turku, Finland

**Keywords:** dendritic cells, lymphatics, lymphatic vessels, traffic, migration, immunosuppression

## Abstract

Clever-1 also known as Stabilin-1 and FEEL-1 is a scavenger molecule expressed on a subpopulation of anti-inflammatory macrophages and lymphatic endothelial cells (LECs). However, its role in regulating dendritic cell (DC) trafficking and subsequent effects on immunity have remained unexplored. In this study, we demonstrate that DC trafficking from the skin into the draining lymph nodes is compromised in the absence of Clever-1. By adoptive transfer approaches we further show that the poor trafficking is due to the impaired entrance of DCs into afferent lymphatics. Despite this, injections of ovalbumin-loaded DCs into the footpads induced a stronger proliferative response of OT II T cells in the draining lymph nodes. This could be explained by the increased MHC II expression on DCs and a less tolerogenic phenotype of LECs in lymph nodes of Clever-1 knockout mice. Thus, although fewer DCs reach the nodes, they are more active in creating antigen-specific immune responses. This suggests that the DCs migrating to the draining lymph node within Clever-1 positive lymphatics experience immunosuppressive interactions with LECs. In conclusion, besides being a trafficking molecule on lymphatic vasculature Clever-1 is immunosuppressive towards migrating DCs and thus, regulates the magnitude of immune responses created by incoming DCs in the draining lymph nodes.

## Introduction

To initiate an effective adaptive immune response or tolerance, dendritic cells (DCs) among other leukocytes, migrate from the periphery to the draining lymph nodes (dLNs) *via* the afferent lymphatic vessels. In contrast to other cells, only lymphocytes can leave the nodes *via* the efferent lymphatics and exert their immune functions elsewhere in the body. DC trafficking from the periphery to the draining lymph nodes through lymphatic vessels and their ability to carry and present antigens in the node is an essential element in the induction of the immune response within the lymph nodes (1–4). It has been shown that chemokines and their receptors, adhesion molecules and integrins expressed on lymphatics are involved in DC transmigration into lymphatic vessels and their migration towards the dLNs. To date, a few molecules on lymphatic endothelial cells (LECs) have been identified to be responsible for dendritic cell migration within the afferent lymphatics. They include sphingosine 1 phosphate, CD31, CD99, Semaphorin 3A, Podoplanin, LYVE-1, and chemokines such as CCL21 and CXCL12. In inflammatory conditions, also ICAM-1, VCAM-1, ALCAM, D6, and CX3CL1 on lymphatics contribute to the trafficking of dendritic cells (5–8). In addition to DCs, LECs of dLNs play an essential role in regulating both tolerance and immunity. Even though LECs do not express co-stimulatory molecules such as 4-1BBL, CD86, and CD80, they endocytose antigens, cross-present MHC I antigens and express PD-L1 and MHC II molecules (4).

Clever-1 is a scavenger receptor expressed on both afferent and efferent arms of the lymphatic vasculature and it has been demonstrated to be involved in lymphocyte and cancer cell trafficking within the lymphatics (9–11). Moreover, Clever-1 mediates trafficking of B cells and CD8 positive T cells into the splenic red pulp, but not significantly to other lymphatic organs *via* blood vasculature in normal conditions (12). However, it is also induced in the flat walled endothelium at sites of inflammation and in certain cancers (9, 12, 13). However, its role in DC trafficking from the periphery into the dLN *via* the lymphatic vessels as well as its role on lymphatics in modulating immune responses in the dLN have not been previously explored. In this work, we utilized Clever-1 KO mice and tested DCs trafficking *via* skin draining lymphatic vessels and compared that to the trafficking of DCs in wild type (WT) mice. Moreover, we studied antigen-specific immune responses in the dLNs in a delayed-type hypersensitivity (DTH) model. The Clever-1 KO mice showed impaired DC transmigration into the skin draining lymphatics that subsequently resulted in lower numbers of DCs in the dLNs. Despite impaired DC trafficking, the antigen-specific immune response was normal in KO mice. We also analyzed the characteristics of the lymphatics of dLNs in Clever-1 KO mice and their WT controls at steady-state and inflammation using RNA sequencing. LECs of LNs lacking Clever-1 has a more proinflammatory phenotype than their WT controls at steady state, explaining the normal level of the antigen-specific immune response, despite impaired DC trafficking into the dLNs.

## Materials and Methods

### Study Design

This study examined the role of Clever-1 on lymphatic endothelium in regulating the traffic of DCs into the draining lymph nodes and the consequences of Clever-1-dependent interaction between DCs and lymphatics on the activity status of the migrating DCs. Both adoptively transferred and endogenous DCs and Kikume and CD11c^+^-YFP^+^ reporter mice were used in the migration studies and entrance of DCs into the lymphatics was visualized using advanced microscopy. T cell responses against footpad injected ovalbumin were analyzed in the dLNs. To find the reason for the aberrant behavior of DCs with lymphatics, RNAseq analyses were performed for lymphatic endothelial cells of Clever-1 WT and KO mice. Moreover, the contribution of Clever-1 on lymphatics and blood endothelium was analyzed in OXA-induced skin inflammation (contact-induced hypersensitivity model).

### Mice

Clever-1 KO mice with a mixed C57BL/6N and 129SvJ background were generated as previously described (10) and used with their WT controls (both sexes in a randomized fashion) at an age between 6 and 12 weeks. DsRed mice [B6.Cg-Tg(CAG-DsRed*MST)1Nagy/J] carrying a red fluorescent reporter protein as a transgene were from the Jackson Laboratory (stock number 006051). Balb/C mice were from Janvier and Charles River, CD11c^+^-YFP^+^ mice from Jackson and Kikume mice (14) from Prof. Masayuki Miyasaka, Osaka University. All animal experiments were approved by The Finnish Act on Animal Experimentation (62/2006); animal license number 5762/04.10.07/2017 and 12537/2020).

### Immunohistochemistry

To examine the cell populations of dLN and Clever-1 expression, frozen dLN sections were stained with antibodies specific for CD3 (BD Biosciences), B220 (eBioscience), MHCII (eBioscience), LYVE-1 (eBioscience), Clever-1 (9–11, recognizing both mouse and human Clever-1 (15). To evaluate the structure of lymphatic vessels as well as entry of DCs into the lymphatics, ear sheets were stained with antibodies specific for Podoplanin (BioLegend, 127410) and LYVE-1 (eBioscience). Thereafter, the entire sections were imaged using 3i spinning disk confocal microscopy (Carl Zeiss SAS, Jena, Germany), Zeiss LSM 780 or LSM 880 confocal microscope equipped with ZEN imaging software (both from Carl Zeiss SAS, Jena, Germany). Images were analyzed using ImageJ software (Rasband, W.S., ImageJ, U.S. National Institutes of Health, Bethesda, MD, USA) or Imaris 8 (Bitplane Inc).

### Microlymphography

To evaluate the function of lymphatic vessels in WT and KO mice tetramethylrhodamine (TRITC)–dextran (molecular weight, 2 million Da; Sigma) was intradermally injected into the ear tip and its distribution was instantly imaged under fluorescence microscopy (Leica).

### FITC Skin Painting

One percent FITC in 1:1 acetone: dibutyl phthalate was painted on the dorsal side of the ears of WT and KO mice. Ear-draining auricular LNs were collected for analysis 20 and 48 h after the painting. dLNs were digested for 30 min in 100 micrograms/ml DNase I and 0.5 mg/ml Collagenase P at 37°C. EDTA was added for the final 5 min incubation. The single-cell suspensions were stained for flow cytometry: CD45-BV650, CD103-PE, CD11c-PerCP-Cy5.5 or CD11C-VB421, CD11b-eFluor450 or CD11b-APC-Cy7, and MHC II-PE-Cy7 or MHC II- PerCP-eFluor 710 (all from BD Biosciences) for 20 min, washed and fixed with 4% PFA. Thereafter, the cells were washed with 0.5% saponin buffer and stained with Langerin-Alexa Fluor 647 (Dendritics, 929F3.01) for 20 min at RT. The samples were measured with LSR Fortessa flow cytometer (BD) and analyzed with FlowJo software (Treestar). Dendritic cell populations were defined among CD11c+MHCII^hi^ events as follows: Langerin^+^CD103^-^ (Langerhans cells), Langerin^+^CD103^+^ (CD103^+^ dermal dendritic cells, DDC), Langerin^-^CD11b^+^ (CD11b^+^ DDC) and Langerin^-^CD11b^-^ (double negative DDC).

### Dendritic Cell Injections

Bone marrow cells from CAG-DsRed reporter mice were cultured with recombinant murine GM-CSF (PEPROTECH) and matured with LPS (Sigma) as previously described (16). 1 ✕ 10^5^ fluorescent DCs in 25 µl PBS were injected subcutaneously into footpads of WT and KO mice. Mice were sacrificed 20 h after the injections and single-cell suspensions of the draining lymph nodes were stained for flow cytometry: CD45-PE, CD11c-FITC and MHCII-PE-Cy7 (all from BD Biosciences) for 20 min on ice. Samples were analyzed as described above. Adoptively transferred DCs were defined as follows: CD45^+^CD11c^+^MHCII^hi^DsRed^+^.

For visualizing the trafficking of adoptively transferred BMDCs in the ear dermis, 2.5 ✕ 10^5^ matured CFSE-labeled BMDCs were intradermally injected into the ear of WT and KO mice followed by application of 2% OXA. One day later, the mice were sacrificed, dorsal and ventral portions of the ear were separated, fixed with 4% PFA, permeabilized with 0.1% Triton and stained with conjugated primary antibodies against Lyve-1 (eBioscience) and Podoplanin (BioLegend).

Confocal Z-stack images of whole-mount ears were acquired using 3i spinning disk confocal microscopy (Carl Zeiss SAS, Jena, Germany) or LSM 880 confocal microscope equipped with ZEN imaging software (Carl Zeiss SAS). To quantify the number of luminal DCs, 3D images were constructed by using Imaris 8 (Bitplane Inc) and the number of FITC^+^ DCs transmigrated inside the lumen versus those located in the vicinity of lymphatic vessels were counted.

### Split Ear Assays

Dorsal and ventral portions of the ear were manually separated and dorsal portions were discarded. CD11c^+^-YFP^+^, DsRed^+^ or WT LPS-matured BMDCs were generated as described and purified with the pan-dendritic cell isolation kit (Miltenyi). Matured BMDCs were incubated on the ventral portion of the ear for 20 min. Thereafter, the non-adherent cells were washed away and incubation was continued in fresh medium for 2 h at 37°C in 5% CO2. The ear sheets were fixed with 4% PFA and stained with conjugated primary antibodies against Podoplanin (BioLegend) and LYVE-1 (eBioscience). The whole-mount ear sections were imaged using 3i spinning disk confocal microscopy (Carl Zeiss SAS), Zeiss LSM 780 or LSM 880 confocal microscope equipped with ZEN imaging software (both from Carl Zeiss SAS). The length of lymphatic vessels, as well as the number of DCs colocalized with the lymphatic vessels, were counted using ImageJ software.

### Ovalbumin-Specific T Cell Response Model

To evaluate OT II T cell response in the presence of a soluble antigen, 5.5 ✕ 10^4^ LPS activated WT BMDCs were loaded with 2.5 µg/ml of OVA_323-339_ peptide (SSINFEKL) and after vigorous washes injected into the footpad of Clever-1 WT and KO mice. One day later, OT II splenocytes were first labeled with 5 µM Vybrant CFDA SE Cell Tracer (CFSE; Thermo Fisher Scientific) according to the manufacturer’s instructions. 5 ✕ 10^6^ CFSE-labeled splenocytes in 200 µl of PBS were intravenously injected into the WT and KO mice. The popliteal draining LNs and inguinal distal LNs were collected 48 h later, single-cell suspensions were prepared and OT II T cell proliferation was evaluated by measuring CFSE dilution among the OT II CFSE labeled cells using flow cytometry. Single-cell suspensions were stained for flow cytometry: CD45-APC-Cy7, CD3-Alexa Fluor 647, B220-BV421, and CD4-APC-cy7 (all from BD Biosciences) for 20 min on ice. Samples were measured and analyzed as described above.

To analyze the uptake of OVA in the dLN, 5 mg/ml of DQ-OVA (Thermo Fisher Scientific) was subcutaneously injected into the hind footpad of WT and KO mice. The draining popliteal LNs were collected 90 min later and embedded in OCT blocks. Frozen sections of LNs were analyzed for the presence of digested DQ-OVA that emits in the green spectrum.

### Contact Hypersensitivity Model

Mice were first sensitized on the shaved belly (50 µl) and each paw (5 µl) with 2% OXA (4-ethoxymethylene-2-phenyl-2-oxazoline-5-one; Sigma-Aldrich) in acetone/olive oil (4:1 volume/volume). Five days later, the mice were challenged by topical application of 10 µl of 1% OXA solution on each side of the ear. The ear measurements were performed from 1 to 8 days after the OXA challenge. Non-Inflamed control and inflamed ear-skin were collected and digested with whole skin dissociation kit (Miltenyi Biotec) according to the manufacturer’s instructions. The single-cell suspensions were stained for flow cytometry: CD45-eFluor 700, CD3-FITC, CD4-APC-Cy7, CD8a-BV650, CD11c-BV421, MHC II-PerCP-eFluor 710, CD103-Bv510, CD25-APC, CD11b-PE (all from BD Biosciences), and CD25-APC (eBioscience) for 20 min on ice. For Langerin staining, samples were fixed with 4% PFA, washed with permeabilizing buffer (eBioscience) and finally stained with Langerin-Alexa Fluor 647 (Dendritics) for 20 min at RT. For Foxp3 staining, samples were fixed and permeabilized with Transcription Factor Staining Buffer Set (eBioscience) according to the manufacturer’s instruction and stained with Foxp3-PE-CF594 (BD Biosciences). Samples were measured and analyzed as described above.

In another set of CHS experiments, mice were sensitized with 2% OXA as described above. Five days later, single-cell suspensions of skin-draining axillary and inguinal LNs (10 million cells in 160 µl of PBS) were injected *via* the tail vein of the recipients. Alternatively, T cells were isolated by T cell isolation kit (EasySep™) and 10 million isolated T cells in 160 µl of PBS were injected *via* the tail vein. One hour later, the mice were challenged by topical application of 10 µl of 1% OXA solution on each side of the ear. The ear measurement was performed 1 and 2 days after the OXA challenge.

### Bone Marrow Transplantations

To generate bone marrow chimaeras, C57BL/6N Clever-1^-/-^ and their WT control were irradiated twice with 5 Gy (Faxitron Multirad 350) using 3-h intervals followed by injections of 10 X 10^6^ bone marrow cells collected either from KikGR photoconvertible transgenic or CD11c^+^-YFP^+^ reporter mice. To track the photoconverted cells in Kikume chimaeras, the shaved belly was irradiated with UV light followed by application of 2% OXA (Sigma). One day later, the axillary draining LNs were stained with above-mentioned antibodies for flow cytometry analyses.

### siRNA Silencing of Clever-1 on Human Dermal Lymphatic Endothelial Cells

HDLECs were obtained from Promocell and cultured in MV2 medium (C-22022, Promocell). For silencing, 30 000 cells/well were seeded on fibronectin-coated 12-well plates. One day later, the cells were silenced for Clever-1 by lipofection with Lipofectamine RNAiMAX (ThermoFisher Scientific) and using 15 nM siRNA (ON-TARGETplus siRNA, human STAB1, J-014103-08-0020, Dharmacon) or a control construct (ON-TARGETplus Non-targeting Control Pool, D-001810-10-20, Dharmacon).

### qPCR

RNA from siRNA-silenced and control cells was extracted with the NucleoSpin RNA kit from Macherey-Nagel and the cDNA was generated with the SuperScript VILO cDNA Synthesis Kit (ThermoFisher Scientific). Clever-1 expression was determined by using the UPL system with probe #74 and primers (left: cac atg tgc caa gaa gat cc; right: cac agc gtg cca aag aaa c). For calculating gene expression, the 2^(-ddCT)^ method was used.

### Generation of Human Monocyte-Derived DCs

moDCs were generated by extracting monocytes from buffy coats obtained from the Finnish Red Cross Blood Service (permit number 22/2018). This was done first by extracting peripheral blood mononuclear cells with gradient centrifugation (Ficoll-Paque PLUS, GE Healthcare), before extracting monocytes with CD14 MicroBeads from Miltenyi Biotec. The purified monocytes were then cultured for 6 days in RPMI 1640 medium (Sigma) that contained 10% FCS (Sigma), 4nM GlutaMax (Gibco, ThermoFisher Scientific), 100 U/ml penicillin, 100 µg/ml streptomycin, 500 U/ml GM-CSF and 350 U/ml IL-4 (both from Peprotech). On day 3, half the medium was replaced with fresh medium.

### Co-culture of Human moDCs and HDLECs

In 12-well plates, 500 000 moDCs were co-cultured together with confluent Clever-1 silenced or control-treated HDLECs for 2 days in MV2 medium. After the co-culture, the activation-status of the moDCs was evaluated by staining them with antibodies against CD40, CD83 and MHC class II (all from BD) or respective control antibodies. The cells were run with LSR Fortessa flow cytometer (BD) and analyzed with FlowJo (v10, TreeStar).

### Mixed Leukocyte Reaction Assay

T cells were extracted from the blood of healthy volunteers by using T cell isolation kit (EasySep™) and labeled with 1 µM Vybrant CFDA SE Cell Tracer (CFSE; Thermo Fisher Scientific) according to the manufacturer’s instructions. To assess T cell proliferation, 100,000 T cells together with 10,000 moDCs were added to wells of a 96 well plate that contained either Clever-1 silenced HDLECs or their control-treated counterpart. The cells were cultured in a mix of 50% MV2 medium and 50% RPMI 1640 (containing 10% FCS, 4nM GlutaMax, 100 U/ml penicillin, and 100 µg/ml streptomycin). After 7 days of co-culture, the T cells were stained for flow cytometry: CD8-APC and CD4-PE (all from BD Biosciences) for 30 min on ice. Samples were measured and analyzed as described above.

### RNA Sequencing

Popliteal and brachial LNs of the WT and KO mice at steady-state and 1 day after s.c injection of OVA (1 mg/ml) (1:1) (EndoGrade) emulsified in incomplete Freund’s adjuvant (Sigma) into the footpads were collected and digested to obtain single-cell suspensions. Thereafter, the suspensions were depleted from hematopoietic cells using mouse anti-CD45 microbeads (Miltenyi Biotec). The enriched cells were stained with LIVE/DEAD fixable near-IR dead cell stain (Thermo Fisher Scientific) and conjugated primary antibodies against mouse Podoplanin (BioLegend) and CD31 (BioLegend). Live lymphatic endothelial cells (CD45^-^ CD31^+^ Podoplanin^+^) were sorted into TRIsure (Bioline) using Sony cell sorter, equipped with a 100 µm tip.

Total RNA was extracted from the LECs with RNeasy Plus Micro kit (QIAGEN) and the libraries were prepared using SMART-Seq v4 Ultra Low Input RNA Kit (Takara) and Illumina Nextera XT DNA Library Preparation protocols (Illumina). Sequencing was performed with the Illumina HiSeq 3000 instrument using single-end sequencing chemistry with 50-bp read length at the Finnish Functional Genomics Centre, University of Turku and Åbo Akademi and Biocenter Finland.

### Bioinformatics

The raw sequencing data were uploaded to the BaseSpace Sequence Hub (Illumina) as FASTQ files for further analysis. The quality control was performed using the FastQC application of BaseSpace and subsequently, the sequences were aligned against the mouse reference genome mm10 (UCSC, RefSeq gene annotation) with the RNA-Seq Alignment application, which uses the STAR aligner for read mapping and salmon for quantification of reference genes and transcripts. The differences in gene expression between the samples were identified with the RNA-Seq Differential Expression application using DESeq2. The genes exhibiting a fold change (FC) >2 (log2ratio ≥1 and ≤-1) and q-values (FDR) < 0.05 were selected as differentially expressed genes (DEG). The RNA sequencing data is in the Gene Expression Omnibus (GEO) database under the accession number GSE148730.

Further analyses of the data were performed using QIAGEN Ingenuity Pathway Analysis (QIAGEN IPA) and the Venn diagrams were calculated and drawn with the Venn diagram tool at http://bioinformatics.psb.ugent.be/webtools/Venn.

### Multiplex Analyses

Skin ear and ear-draining auricular LNs from WT and KO mice at steady-state and 1-day OXA (Sigma) treated, 1- and 2-days CHS OXA-challenged mice were collected. Tissues were lysed with ReadyPrep™ Protein Extraction Kit (Bio-Rad) and stored at -70°C. Protein concentration was determined with the DC Protein Assay (Bio-Rad) and 12.5 µg of total protein was used for the multiplex. The multiplex analysis was performed with the Bio-Plex Mouse cytokines 23-plex assay (Bio-Rad), according to manufacturer’s instructions. Bio-Plex 200 system (Bio-Rad) was used to analyze the samples.

### Statistical Analyses

An unpaired two-tailed Student’s t-test with Welch’s correction (when variances were significantly different) and the Mann-Whitney U test were used for statistical analyses unless stated otherwise. For multiple comparisons, one-way ANOVA was used with Tukey’s test. Data points determined to be significant outliers by Grubbs’ test were not included in statistical analyses. P < 0.05 was considered statistically significant (*P < 0.05, **P < 0.01, and ***P < 0.001).

## Results

### Clever-1 Is Expressed in Mouse Skin Lymphatic Vessels

**Figure 1:**
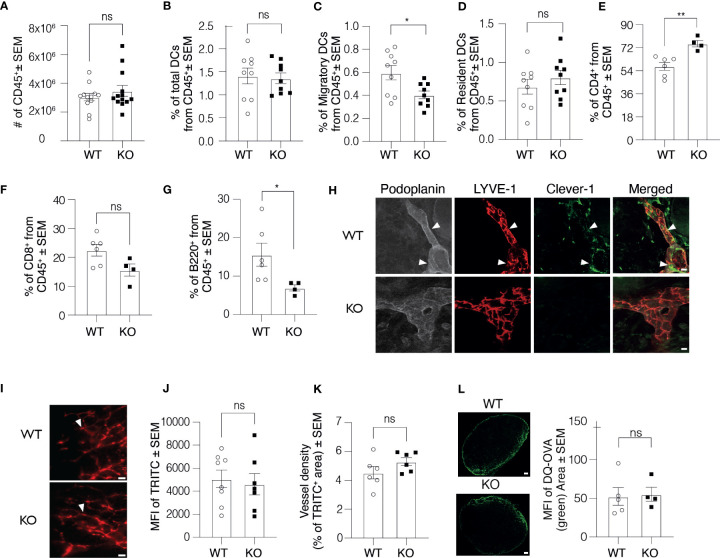
Clever-1 KO mice have morphologically and functionally normal lymphatic vessels. **(A–G)** Flow cytometry analysis of digested auricular dLNs from WT and KO mice, showing the absolute number of leukocytes **(A)**, the percentage of the total number of DCs **(B)**, migratory DCs **(C)**, resident DCs **(D)**, CD4^+^ T lymphocytes **(E)**, CD8^+^ T lymphocytes **(F)**, and B220^+^ B lymphocytes **(G)**; each dot represents one mouse. **(H)** Confocal microscopy images of whole-mount staining of the ear dermis of WT and KO mice stained with antibodies against Podoplanin (grey), LYVE-1 (red) and Clever-1 (green). Arrowheads point to the triple-positive lymphatics. **(I–J)** Fluorescence-microlymphography of the WT and KO ear after intradermal injection of 2 MDa TRITC-dextran **(I)**, and quantification of fluorescence intensity **(J)** and vessel density **(K)**. Arrowheads point to lymphatics; each dot represents one ear. **(L)** Confocal images and quantification of DQ-OVA distribution (green) in the dLNs of WT and KO mice 90 min after subcutaneous injections. Data are presented as mean ± SEM. *P < 0.05 and **P < 0.01, two-tailed Student’s t-test. Scale bars **(H)** 10 µm, **(I)** 300 µm, and **(L)** 200 µm. ns, non-significant.

During the course of studies using Clever-1 KO mice, we have observed cell composition differences in their lymphoid organs compared to WT mice. To investigate the contribution of different immune cells to this phenomenon and the cause of this difference, we first performed a thorough assessment of the immune cell composition in the skin dLNs of Clever-1 KO mice. Flow cytometry analyses of skin dLNs revealed that the absolute number of leukocytes (CD45^+^), total numbers of DCs (CD11c^+^MHCII^+^) and resident DCs (CD11c^+^MH II^int^) were comparable between the WT and KO mice, but significantly less migratory DCs (CD11c^+^MHCII^high^) were found in the dLNs of KO mice (**Figures 1A–D**). The KO mice also had a significantly higher number of CD4^+^ T cells and lower number of B cells, while the numbers of CD8^+^ T were similar (**Figures 1E–G**). Clever-1 is expressed by sub-capsular, lymphatic and medullary sinuses in the LNs but absent from DCs and lymphocytes (**Supplementary Figure 1A** and IMGEN; http://rstats.immgen.org/Skyline/skyline.html). It was, however, not clear to what extent Clever-1 is expressed in the most peripheral afferent lymphatic vessels residing in the skin that could explain the reduced migratory DC population in the dLNs. Indeed, we found a patchy staining pattern of Clever-1 on a subset of LYVE-1^+^Podoplanin^+^ lymphatics in the mouse skin (**Figure 1H**). To further evaluate, whether Clever-1 is expressed on the capillary or collecting lymphatic vessels, we stained the ear sections for Prox-1, Podoplanin, LYVE-1 and Clever-1. Clever-1 is expressed on both LYVE-1^+^ capillary and LYVE-1^-^ collecting lymphatic vessels **(** **Supplementary Figure 1B** **)**. To directly evaluate the functionality of the skin lymphatic vessels in WT and KO mice, we performed microlymphography using TRITC-dextran and quantified the fluorescence intensity and the area of TRITC^+^ lymphatic vessels. No major differences were found in the uptake and drainage of TRITC-dextran *via* the cutaneous lymphatics in KO mice indicating that the vessels were functionally normal (**Figures 1I–K**). To further test the influx of antigens into dLNs, we used DQ-OVA as a tracer molecule. DQ-OVA is a self-quenched fluorochrome, which only upon ingestion by phagocytes emits fluorescence in the green spectrum. We injected DQ-OVA into the footpad of WT and KO mice and 90 min later measured the distribution of the green fluorescence signal in the draining popliteal LN. The signal in the LN of KO mice and their WT controls was comparable, indicating an efficient and comparable antigen delivery system in both mice (**Figure 1L**). We further examined the morphology of lymphatic vessels in Clever-1 KO mice by quantifying the LYVE-1^+^ area in whole-mount ear images. Lymphatic vessels in WT and KO mice appeared to be morphologically normal and no differences were found in the area that was covered by LYVE-1^+^Podoplanin^+^ lymphatics (**Supplementary Figures 1C, D** **)**. To exclude the possibility that the number of DCs in the skin would be the reason for the lower number of migratory DCs in the dLNs of KO mice, we quantified the number of DCs and the total number of CD45^+^ cells in the ear skin of WT and KO mice. **T**he total number of DCs and the number of CD45^+^ cells in the ear skin in WT and KO mice were comparable at steady state (**Supplementary Figures 1E, F** **)**.

### Dendritic Cell Trafficking *via* Lymphatics into the Skin dLNs Is Impaired in Clever-1 KO Mice

**Figure 2:**
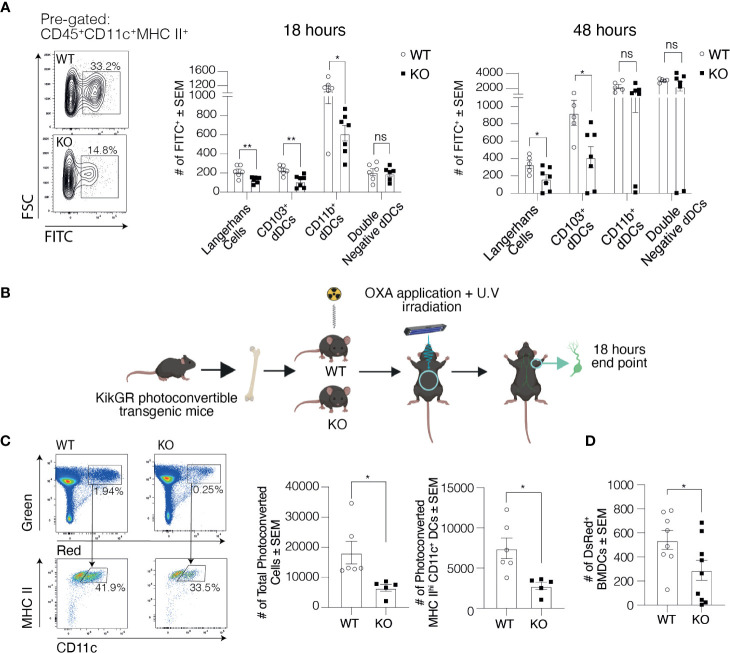
Trafficking of DCs into the skin draining LNs is compromised in the absence of Clever-1. **(A)** Representative dot plots and quantification of FITC positive DCs in the ear-draining auricular LNs at 20 and 48 h after FITC painting. **(B)** Schematic presentation of the generation of KikGR (Kikume) photoconvertible transgenic bone marrow chimaeras. **(C)** Representative dot plots and quantification of flow cytometry analyses of the total number of photoconverted cells (green-red) and migratory DCs (green-red; CD45^+^CD11c^+^MHCII^high^) in the axillary LNs of Kikume chimaeras 18 h after UV irradiation and OXA application. **(D)** Flow cytometry analysis of popliteal LNs 20 h after subcutaneous injection of DsRed^+^ BMDCs into the footpad of Clever-1 WT and KO mice. Each data point represents one mouse. Data are presented as mean ± SEM. *P < 0.05 and **P < 0.01, two-tailed Student’s t-test or two-tailed Student’s t-test with Welch’s correction. ns, non-significant.

To explore the cause for the lower number of migratory DCs in the dLNs of Clever-1 KO mice, we first studied the migration of dermal DCs into dLNs 20-h after applying FITC on the ears of WT and KO mice. We observed an overall reduced number of FITC positive CD11c^+^MHCII^high^ cells in the dLNs of KO mice compared to WT controls (**Figure 2A**). Further analysis of the different DC subpopulations showed that the numbers of Langerhans cells (CD11c^+^MHCII^high^CD207^+^CD103^-^) and CD103^+^ dermal DCs (CD11c^+^MHCII^high^CD207^+^CD103^+^) were significantly reduced while no statistically significant difference was seen in the migration of double negative dermal DCs (CD11c^+^MHCII^high^CD207^-^CD11b; **Figure 2A**). The difference remained significant between the WT and KO mice both in Langerhans and CD103^+^ dermal DC subpopulations 48 h after FITC application (**Figure 2A**).

We next took advantage of KikGR photoconvertible transgenic mice (i.e. so-called Kikume mice), in which the migration of endogenous green cells can be followed after they have been photoconverted to red by UV light. We produced Kikume chimaeras by lethally irradiating Clever-1 WT and KO mice and reconstituted their bone marrow with Kikume cells. The belly of the chimaeras was exposed to UV light followed by topical application of 2% oxazolone (OXA; **Figure 2B**). Eighteen hours after conversion significantly less skin-derived photoconverted cells (green-red) had migrated to the axillary dLNs of the KO mice, which were identified to consist mostly of migratory DCs (CD11c^+^MHCII^high^; **Figure 2C**). To further validate these findings, we injected DsRed^+^ bone marrow-derived dendritic cells (BMDC) into the footpad of Clever-1 WT and KO mice and 20-h later harvested the popliteal LNs. In line with previous data, the migration of the transferred DsRed^+^ BMDCs was reduced by 46±21% in KO mice compared to WT mice (**Figure 2D**). Thus, using these three different approaches we could confirm the impaired trafficking of migratory DCs to the dLNs in KO mice.

### Clever-1 Regulates Transmigration of Dermal DCs Into Lymphatic Vessels

**Figure 3:**
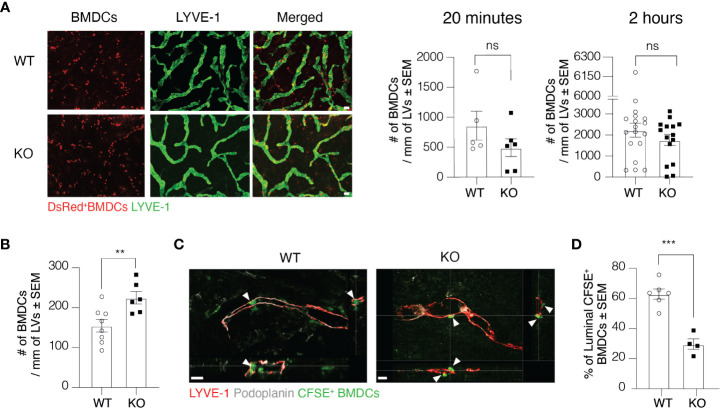
Transmigration of DCs into lymphatic vessels is impaired in the absence of Clever-1. **(A)** Example images and quantification of DsRed^+^ BMDCs on lymphatic vessels (LVs) in dermal ear explants of WT and KO mice at 20 min and 2-h time points. **(B)** Quantification of BMDC adherence on OXA-inflamed ear lymphatics in dermal ear explants of WT and KO at 2-h time point. **(C)** Orthogonal sections of whole-mount ear dermis of WT and KO mice demonstrating the spatial location of DCs to lymphatics (inside vs. outside of the lumen), and **(D)** quantification of the percentages of CFSE^+^ BMDCs inside the lumen 20** h** after their intradermal injection followed by topical application of 2% OXA. Each data point represents one ear. Data are presented as mean ± SEM. **P < 0.01 and ***P < 0.001, two-tailed Student’s t-test. Scale bar **(A)** 100 µm and **(C)** 50 µm. ns, non-significant.

To further investigate at which level Clever-1 deficiency impairs the trafficking of DCs *via* lymphatics, we first employed the so-called split ear model where BMDCs are incubated on the exposed dermis of murine ear sheets to study DC adhesion and migration towards afferent lymphatics in the ear (17). The adherence of BMDCs on afferent lymphatics was comparable between WT and KO mice at 20 min and 2-h time points suggesting that this step was normal and not causing the impaired DC trafficking at steady state (**Figure 3A**). As inflammation affects the production and expression of different cytokines and adhesion molecules, we performed the split ear model on OXA inflamed ears. Against expectations, a greater number of BMDCs adhered on the ear lymphatics of KO mice after 2 h suggesting that the impairment in DC trafficking into the dLNs was not due to blockage of intra-lymphatic migration but rather in the entrance of DCs to lymphatic vessels (**Figure 3B**). We therefore performed whole-mount 3D confocal imaging of intradermally injected CFSE^+^ BMDCs and found that DCs could not enter the lumen of the Clever-1 negative lymphatics as efficiently as they did when Clever-1 was present (**Figures 3C, D**). Quantitative analyses of confocal Z-stack images showed that approximately 60±8,3% of the CFSE^+^ BMDCs that aligned with LYVE-1^+^Podoplanin^+^ lymphatics were inside the vessel lumen in WT mice, whereas, in KO mice the BMDCs were accumulating at the basolateral surface of the lymphatics and only 30±7% of them were inside the lumen of the dermal lymphatics (**Figure 3D**). Thus, the entrance of DCs into the afferent lymphatics is impaired in the absence of Clever-1.

### Lymphatic Clever-1 Regulates the Activity of DCs to Trigger Immune Response

**Figure 4:**
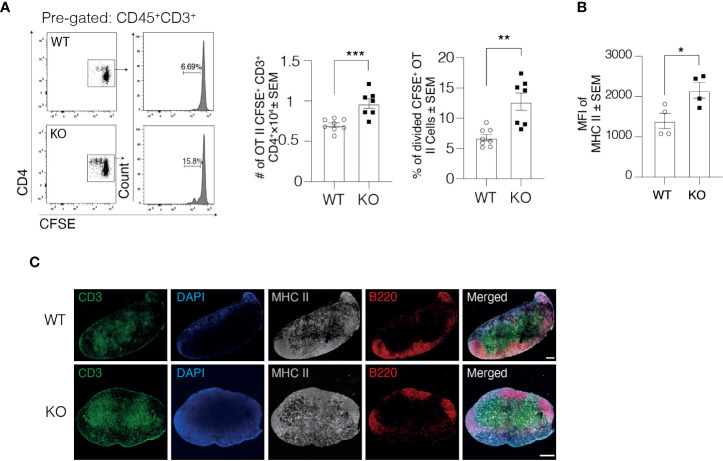
DCs in Clever-1 KO mice have improved antigen presentation to support T cell activation. **(A)** Flow cytometry plots and quantification of the total number of OT II CFSE^+^ CD45^+^CD3^+^CD4^+^ T cells and the percentage of divided cells among donor OT II CFSE^+^ CD45^+^CD3^+^CD4^+^ T cells in the draining popliteal LN of WT and KO mice, 48 h after the i.v. injection of CFSE labeled OT II splenocytes. **(B)** MHC II expression by DCs recovered from the draining popliteal LNs 18 h after OVA (emulsified in incomplete Freund’s adjuvant) injection and analyzed by flow cytometry; each dot represents 2 pooled animals. **(C)** Fluorescence staining of the dLNs of KO mice and their WT controls: B cells (anti-B220, red), T cells (anti-CD3, green), DCs and macrophages with anti-MHC II (grey) and nucleus with DAPI (blue). Data are presented as mean ± SEM. *P < 0.05, **P < 0.01, and ***P < 0.001, two-tailed Student’s t-test or two-tailed Student’s t-test with Welch’s correction. Scale bars 200 µm.

Due to impaired DC trafficking, we next asked whether antigen-specific immune responses in the dLN were affected by the lack of Clever-1. In this set of studies, we first injected ovalbumin (OVA) peptide-loaded DCs into the footpad of WT and KO mice and 1 day later, OVA-specific CFSE labeled CD4^+^ T cells from OT II mice were intravenously injected into the mice. The OVA-specific response was analyzed 2 days later by measuring T cell proliferation by CFSE dilution in the draining popliteal LNs (**Figure 4A**). Quantification of OT II cells showed that the total number of CFSE^+^ CD3^+^ CD4^+^ OT II cells in the draining popliteal LNs of KO mice was 37.9 ± 8% higher than in WT mice, indicating a stronger antigen-specific immune response in the draining popliteal LNs of KO mice (**Figure 4A**). Further analysis of the proliferated OT II cells showed a 87.32 ± 22% increase in the percentage of divided OT II cells in the draining popliteal LNs of KO mice compared to their WT controls.

Engagement of MHC II expressed on DCs with T cell receptor on CD4^+^ T cells results in T cell activation. To investigate the underlying mechanisms for the higher antigen-dependent immune response, we analyzed the expression level of MHC II on DCs 1 day after injecting OVA into the footpad of WT and KO mice. The DCs in KO mice expressed higher levels of MHC II but not CD40 in the draining popliteal LNs compared to WT mice (**Figure 4B** and **Supplementary Figure 1G**), indicating that DCs in the dLNs of KO mice could most likely activate more T cells on a per-cell basis. Comparable findings were observed in a non-manipulated situation where the MHC II signal was markedly higher within the LNs of KO mice (**Figure 4C** **)**. These results together with the normal flux of antigen to the draining LNs in KO mice presented in **Figure 1** indicate that the delivery of free antigens to the dLNs is normal in KO mice but DCs that have migrated through Clever-1 negative lymphatics are more activated based on increased MHC II expression.

As mouse lymphatics do not retain Clever-1 expression in culture, we used human LECs to further study, whether the strong antigen-specific immune response is due to the lack of Clever-1 expressed by LECs. In this set of experiments, we first silenced Clever-1 HDLECs by siRNA. Clever-1 mRNA level reduced by 74% and protein level by 65% compared to control siRNA-transfected cells **(** **Supplementary Figure 4A** **)**. To further validate, whether the more activated phenotype of DCs is LEC-dependent, we cultured monocyte-derived DCs with Clever-1 or control siRNA transfected HDLECs for 2 days and analyzed the expression of MHCII, CD40 and CD83 by DCs. In line with our mouse data, MHCII expression was upregulated on DCs, when cultured with Clever-1 siRNA treated HDLECs. In addition to MHCII, expression of CD40 and CD83 were increased on DCs, when cultured with Celver-1 siRNA treated HDLECs. **(** **Supplementary Figure 4B** **)**. Next, we performed mixed leukocyte reaction (MLR) assays, in which monocyte-derived DCs were incubated with allogenic T cells in the presence of Clever-1 silenced HDLECs or siControl treated HDLECs for 7 days. In line with our *in vivo* mouse data, T cell proliferation in the presence of Clever-1 silenced HDLECs increased by approximately 17.65 ± 7% (**Supplementary Figure 4C**). These results suggest that the function of DCs is fine-tuned by Clever-1 on LECs.

## Clever-1 Negative Lymphatics Overexpress Genes Regulating Immune Response and Downregulate Those Involved in Transmigration

**Figure 5:**
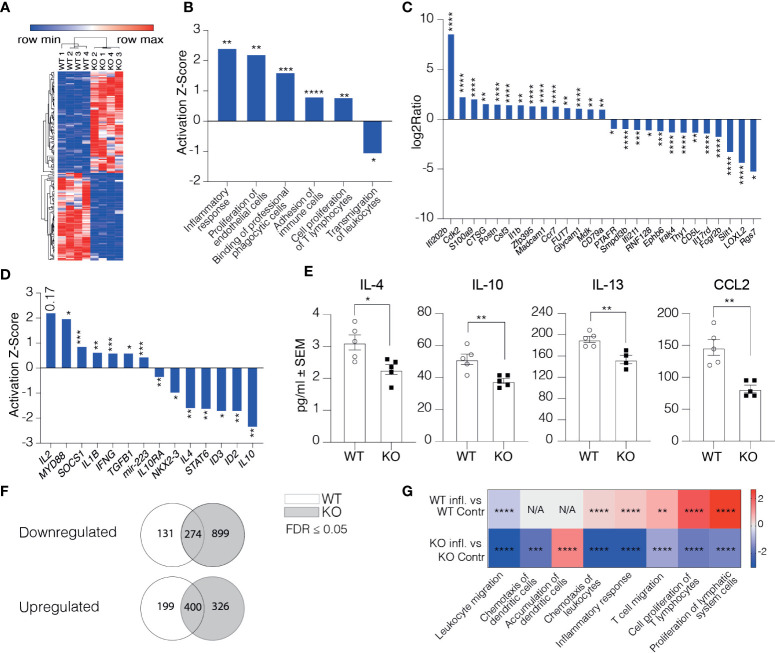
Clever-1 supports lymphatic endothelial cell tolerance. **(A)** Heat map of the gene expression differences in LECs obtained from LNs of Clever-1 KO and their WT controls. Unsupervised hierarchical clustering was performed on the log 2 ratio of significantly (FDR < 0.05) up- (red) and down-regulated (blue) genes. **(B)** Altered pathways in KO LECs compared to WT LECs analyzed by using the disease and Bio-function in IPA. **(C)** Genes in KO LECs (Fold Change ≥ 2) belonging to the altered pathways shown in **(B)**. **(D)** IPA Upstream regulator analysis of DEGs between WT and Clever-1 KO LECs at steady state. **(E)** Cytokine levels in the dLNs at steady state. **(F)** Venn diagrams summarizing differentially expressed genes 1 day after footpad injection with OVA emulsified in incomplete Freund’s adjuvant. **(G)** Most significantly altered pathways in inflamed and non-inflamed LECs of WT and KO mice analyzed by using the disease and Bio-function in IPA. Each dot represents one mouse. Data and statistical significance in **(B, D, G)** were analysed by using IPA, *P < 0.05, **P < 0.01 and ***P < 0.001 ****P < 0.0001. Data and statistical significance in **(C)** were analysed by using Illumina, * FDR < 0.05, **FDR < 0.01 and *** FDR < 0.001 **** FDR < 0.0001. Data in **(E)** is expressed as mean ± SEM. *P < 0.05 and **P < 0.01 , two-tailed Student’s t-test.

To understand how the afferent lymphatics could regulate the phenotype of DCs arriving in LNs, we performed RNA-seq on CD45^-^CD31^+^Podoplanin^+^ cells of the skin-draining LNs from Clever-1 KO mice and their WT controls. All the samples expressed high levels of LEC marker genes, including *Pdpn*, *Pecam-1*, *Prox-1*, and *Flt4* with a comparable expression between the WT and KO mice. The only exception was *Pdpn* that was slightly but statistically significantly lower in KO mice (**Supplementary Figure 2A**). *Stab1* was absent in the Clever-1 KO LECs as expected (**Supplementary Figure 2A**). At steady state, the Clever-1 KO LECs had 162 differently expressed genes (DEG) (FDR ≤0.05, log2ratio ≥1 and ≤-1) of which 75 were downregulated and 87 genes upregulated (**Figure 5A**). To analyze the biological functions of the observed DEGs Ingenuity Pathway Analysis (IPA) software was used. Disease and Bio-function analyses revealed an overrepresentation of genes comprising pathways of immune response regulation, such as inflammatory response and proliferation of T lymphocytes (**Figure 5B**). The most significant genes involved in these pathways were *Ifi202b, Il1b*, *Postn*, *Csf3*, *Fut7*, *Glycam1*, and *Madcam1*, among others (**Figure 5C**). Despite upregulation of pathways related to binding of professional phagocytes and adhesion of immune cells, a pathway related to the transmigration of leukocytes was significantly downregulated in Clever-1 KO LECs (**Figure 5B**). The upstream regulator analytic feature in IPA identified several possible upstream regulators that could explain the observed gene changes (**Figure 5D**). One of them is *Il10*, which is predicted to be significantly inhibited in KO LECs (Z-score -2.36, p=5.8E^-03^). In contrast, *Il2* was predicted to be activated (Z-score 2.213; **Figure 5D**). This indicates that pro-inflammatory signaling cascades were a dominant feature in Clever-1 KO LECs at steady state. Protein analyses of whole LN lysates of WT and KO mice showed reduced levels of anti-inflammatory cytokines IL-4, -10, and -13 as well as CCL2 chemokine, which is in line with the pro-inflammatory gene dominance in the lymphatics of KO mice (**Figure 5E**).

To investigate how the Clever-1 KO lymphatics responded to an inflammatory stimulus, we analyzed the transcriptome of dLN LECs 1 day after injecting OVA emulsified in incomplete Freund’s adjuvant into the footpad of WT and KO mice. WT LECs downregulated 274 and upregulated 400 genes by OVA-CFA administration. The Clever-1 KO LECs showed specific downregulation of an additional 899 and upregulation of 326 genes (**Figure 5F** and **Supplementary Figure 2B**). Interestingly, these changes in KO mice were associated with the downregulation of adaptive immune response and leukocyte migration/extravasation pathways (**Figure 5G** and **Supplementary Figure 2C**), which were opposite to the OVA-induced changes in WT LECs (**Supplementary Figure 2D**). However, the KO LECs upregulated *Ackr2*, *Tlr3* and *Csf2* (**Supplementary Figure 2D**), that is in line in the activation of a pathway related to “accumulation of dendritic cells” in LECs of KO mice (Activation Z-score: 1.406, p=8.22E^-05^) (**Figure 5G**).

## Clever-1 KO Mice Have Low Response to Contact Hypersensitivity Reactions

**Figure 6:**
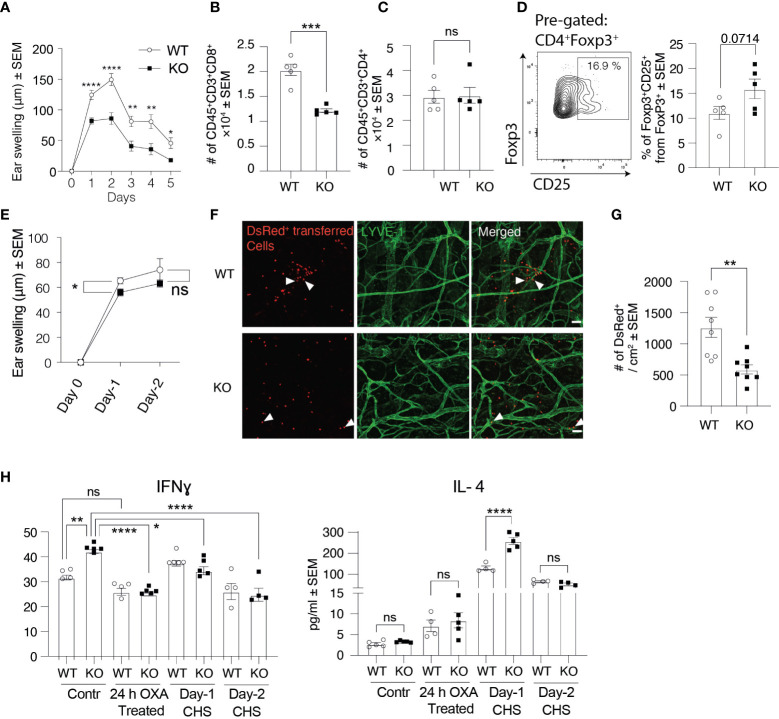
Clever-1 KO mice develop milder inflammatory reactions than their WT controls. **(A)** Ear swelling at indicated time points after oxazolone (OXA) challenge in the CHS model (n≥7 in both KO and WT). **(B–D)** Flow cytometry analyses of digested ear tissue of WT and KO mice, 2 days after the OXA challenge, showing the percentages of endogenous CD8^+^ **(B)**, CD4^+^ **(C)**, and gating and quantification of CD25^+^ Tregs **(D)**. **(E)** Ear swelling at indicated time points after applying OXA on the ears of WT and KO mice followed by adoptive transfer of DsRed^+^ OXA-primed lymphocytes obtained from dLNs of WT mice. (n= 6-26). **(F, G)** Whole-mount confocal imaging **(F)** and quantification **(G)** of the number of DsRed^+^ lymphocytes in WT and KO ear dermis 2 days after applying OXA on the ears. **(H)** Expression of cytokines in the ear skin of Clever-1 WT and KO mice at steady-state, 24 h–OXA treated ears (application of OXA onto the ears 24 h earlier), 1 and 2 days after OXA challenge (CHS). Each data point represents one mouse. Data are presented as mean ± SEM. *P < 0.05, **P < 0.01, ***P < 0.001 and ****P < 0.0001, two-tailed Student’s t-test or two-tailed Student’s t-test with Welch’s correction. Additionally, **(H)** One-way ANOVA with Tukey’s multiple comparison test. **(F)** Scale bar 50 µm and arrowheads point to adoptively transferred DsRed^+^ lymphocytes. ns, non-significant.

Besides being expressed on afferent lymphatics, Clever-1 is also present in efferent lymphatics and is induced to blood vasculature at sites of inflammation. Therefore, we wanted to investigate this whole cascade and chose the contact hypersensitivity (CHS) model for these analyses. In this model, we used OXA as the hapten for belly skin sensitizing and 5 days thereafter challenged the ear skin with OXA. OXA did not dramatically change the expression of Clever-1 on the ear lymphatics (**Supplementary Figure 1H**). Ear thickness measurements at different time points after the challenge showed that Clever-1 KO mice fail to develop a profound CHS response to OXA as their WT controls (**Figure 6A**). Flow cytometry analysis of CHS-inflamed ears at day 2 showed that the number of CD8^+^ T cells in KO ears was 40.3 ± 6% lower than in those of WT mice, while the number of CD4^+^ T cells remained comparable (**Figures 6B–D**). CD8+ T cells mediate skin inflammation by their cytotoxic activity and CD4+ T cells suppress and resolve CHS in inflamed skin (18), providing a possible reason for the impaired OXA-induced CHS response in KO mice. In fact, FACS analyses of KO ears 2 days after OXA challenge showed that there tend to be more CD25 positive regulatory T cells, supposedly a more suppressive population among migratory T regs (19), in KO mice (**Figure 6D**). To analyze whether the reduced CHS response in KO mice was due to impaired lymphocyte egress from the dLNs after the sensitization phase or impaired lymphocyte entrance into the inflamed ear skin, we isolated OXA-primed lymphocytes from dLNs of DsRed^+^ WT mice and adoptively transferred them into WT and KO mice before applying OXA on their ear skin. In line with our previous results, ear swelling was still reduced in KO mice although not that markedly as in the standard model (**Figure 6E**). Furthermore, whole-mount ear imaging showed that the number of DsRed^+^ lymphocytes was significantly reduced in KO mice compared to WT mice (**Figures 6F, G**). These results indicate that both the exit of effector lymphocytes *via* efferent lymphatics from the sensitized lymph node and entrance into the challenged inflammatory ear *via* blood endothelial cells are affected by the absence of Clever-1.

We also assessed different cytokine levels in the ear skin of KO and WT mice at steady-state and inflammation. Intriguingly, pro-inflammatory cytokines, including IFNγ, IL-1α, and IL-17 were significantly higher at steady-state in KO mice compared to their WT control (**Figure 6H**, **Supplementary Figure 3A**). However, inflammation significantly decreased the levels of pro-inflammatory cytokines in KO mice, while the level of anti-inflammatory cytokine IL-4 was significantly higher in KO mice compared to their WT control, 1 day after the OXA challenge (**Figure 6H**, **Supplementary Figure 3A**). These results indicate that Clever-1 deficiency at steady-state causes a more pro-inflammatory microenvironment, while inflammation dampens an excessive pro-inflammatory state to help maintain homeostasis.

**Figure 7:**
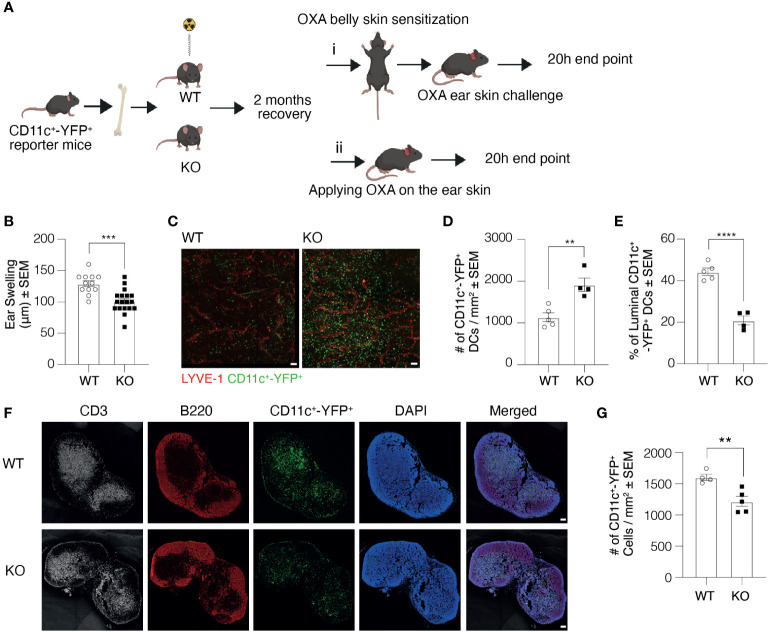
Clever-1 KO macrophages do not contribute to the impaired transmigration of dermal DCs. **(A)** Schematic presentation of the study design illustrating the generation of bone marrow chimaeras and subsequent sensitization with OXA followed by i) challenging the ears with OXA or ii) without previous sensitization. **(B)** Ear thickness of CD11c^+^-YFP^+^ → WT and KO chimaeras 20 h after the OXA challenge (model i). **(C–E)** Whole-mount images of ear dermis **(C)**, and quantification of the total number **(D)**, and the percentage of CD11c^+^-YFP^+^ cells inside the lumen of ear lymphatics **(E)** of CD11c^+^-YFP^+^→WT and KO chimaeras 20 h after topical application of OXA (model ii). **(F, G)** Confocal images of auricular draining LNs **(F)**, and quantifications of the number of CD11c^+^-YFP^+^cells in the auricular LNs **(G)** of CD11c^+^-YFP^+^→WT and KO chimaeras 20 h after topical application of OXA. Each data point represents one mouse. Data are expressed as mean ± SEM. **P < 0.01, ***P < 0.001, and ****P < 0.0001, two-tailed Student’s t-test. Scale bars 100 µm.

As macrophages play a crucial role in DC clustering and migration, and Clever-1 is also expressed on a subset of macrophages, we created bone marrow chimaeras using bone marrow cells acquired from CD11c^+^-YFP^+^ reporter mice and transplanted them to lethally irradiated Clever-1 WT and KO mice (**Figure 7A**). This allowed us to exclude a possible contribution of Clever-1^+^ macrophages in the CHS reaction. In line with previous data, the KO mice created milder inflammation as measured by ear swelling, confirming the role of the endothelium in the impaired CHS reaction of the KO mice (**Figure 7B**). This model also provided us with a possibility to further confirm the role of Clever-1 on afferent lymphatics in DC trafficking. To assess and further validate our results in **Figure 3D** regarding the transmigration of DCs into the lymphatic vessels, the entrance of migratory DCs into the lymphatics was visualized after applying 2% OXA onto the ear skin of the chimeric mice without previous belly sensitization followed by whole-mount confocal imaging of ears 20 h later. Quantification of the number of DCs migrated into the lumen confirmed the finding: although there are more CD11c^+^-YFP^+^ cells in the skin of KO recipients, they do not get access into the lumen of lymphatics as efficiently as in WT mice (**Figures 7C–E**). This was also reflected by the lower number of these cells in the ear draining auricular LNs (**Figures 7F, G**).

## Discussion

In this work, we show that the lymphatic endothelial Clever-1 is a crucial mediator of DC trafficking into the dLNs. Clever-1 displays immunosuppressive imprinting to the DCs during their journey to the LNs, as the DCs migrating *via* the lymphatics lacking Clever-1 trigger higher antigen-specific proliferative responses in the nodes despite their lower numbers. This type of modulation may be central to avoid unnecessarily high immune responses and thus, to keep the immunity at an optimal level.

It has remained unclear how immune cell migration and transport of soluble components within the lymphatics determine the quality and quantity of the immune response (3). It is known that LN LECs can efficiently scavenge and present peripheral antigens on MHCI to induce tolerance by specific deletion of autoreactive CD8^+^ T cells. Moreover, LN LECs can phagocytose, process exogenous antigen and cross-present it to naïve CD8 T cells. However, the dominant expression of co-inhibitory molecules and lack of co-stimulatory molecules by LECs can lead to specific deletion of reactive lymphocytes (20–22). The results of this work strongly suggest that Clever-1 on lymphatic vasculature is an important regulator of DC trafficking and simultaneously an immunomodulator of DC activity when evoking immune responses in the dLNs. Indeed our data showed that knockdown of Clever-1 from human lymphatic endothelial cells leads to a more activated phenotype of DCs in co-cultures. As Clever-1 is also a scavenger rapidly recycling between the plasma membrane and endosomal compartments and being able to scavenge both endogenous and exogenous antigens such as acetylated LDL, placental lactogen, apoptotic cells and bacteria (23–27), it may as well participate in antigen processing.

Clever-1 is expressed rather uniformly both on the afferent and efferent arms of the lymphatics and can play its effects at sites of its expression. In this study, we could demonstrate by both exogenous and endogenous DCs that in the absence of Clever-1 on lymphatics DCs do not enter the afferent lymphatics in a normal fashion. However, as DCs exert their function in the dLNs by presenting antigens and do not normally leave these nodes *via* the efferent lymphatic vessels (28), the contribution of Clever-1 in the efferent lymphatic arm seems mainly to be in the lymphocyte exit from the nodes (9).

**Figure 8:**
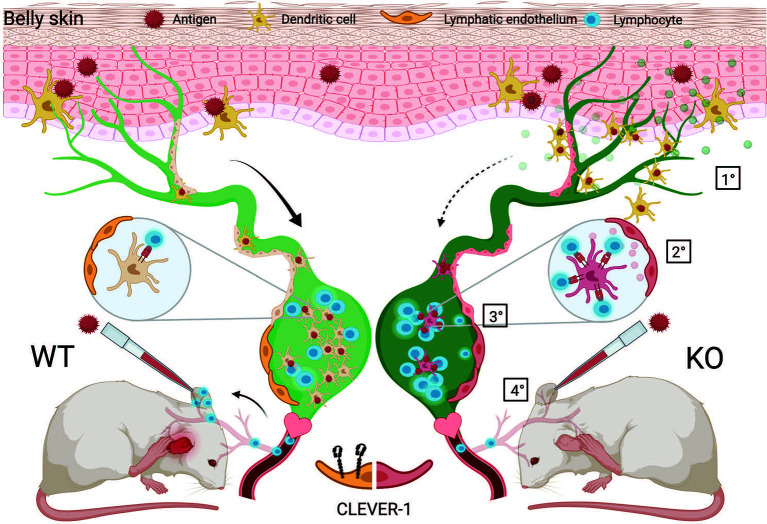
Schematic presentation of the proposed action points of Clever-1. **1)** DC migration from the skin into the draining lymph nodes *via* lymphatic vessels is impaired in the absence of Clever-1 when mice are sensitized with an antigen on the skin. **2)** However, the DCs that end up in the LN show good capacity to activate T cells. This is due to a less tolerogenic interaction of the migrating DCs with Clever-1 deficient lymphatic vessels. **3)** When leaving the lymph node, the activated T cells cannot egress the Clever-1 KO LN as efficiently as in the WT situation and also **4)** the lack of Clever-1 expression on blood vessels at the site of inflammation impairs the homing of T cells into the tissue. This reduces the inflammatory reaction when mice are re-challenged in the ear with the same antigen.

Besides directly investigating DC traffic *via* the afferent lymphatics to the dLNs and consequences in the nodes, we also used a more complex model, namely a contact hypersensitivity model, that allowed us to analyze the summation of the lymphocyte exit from the nodes draining the belly area used for the sensitization and also the entrance *via* the blood vessels to the site of challenge in the ears. Based on normal or even slightly accelerated immune response in the draining lymph nodes as shown in the OVA immunizations and reduced ear swelling at the site of challenge in the contact hypersensitivity experiments, the possible reasons for this phenomenon are: impairment either in the exit of OXA-specific T cells from the draining nodes *via* the efferent lymphatics, their entrance into the ear *via* the blood vessels or both. In this context, it is important to note that although Clever-1 is absent from normal flat walled endothelial cells, it is induced on this type of vessels at sites of inflammation (9, 29). Based on the transfer experiments presented in **Figure 6E** we suggest that both components are involved as exemplified in **Figure 8**. It should also be noted that macrophages deficient of Clever-1 have an increased pro-inflammatory phenotype (30) and could contribute to the increased inflammatory microenvironment that we observed in the LN and ear of Clever-1 KO mice at steady state. This most likely can additively stimulate the increased MHC II expression on migratory DCs.

Immunomodulatory roles for the lymphatic endothelium have been realized during recent years. For example, they can promote tolerance as well as archive and present antigens (31). Lymphatic endothelial cells are known to attenuate inflammation *via* suppression of the maturation of DCs (32). Also, it has been shown that lymphatic endothelial cells can archive, store and present antigens to DCs and prime naïve CD8 T cells into memory cells (33, 34). Moreover, it has been reported that DCs spend considerable time and closely interacting with endothelial cells of afferent lymphatics (35). Moreover, DCs form contacts with intra-lymphatic T cells during their journey indicating that modulation of adaptive immunity occurs already in afferent lymphatics before entering the LNs. In this scenario, Clever-1 deficient lymphatics are not able to down modulate the activity of DCs, which then effectively prepare T cells to provoke an accelerated immune reaction within the dLNs. Indeed, DCs co-cultured with Clever-1 silenced HDLECs, express higher levels of MHCII, and consequently, when used in MLR assay, the proliferation of naïve CD4^+^ T cells significantly increases. These data are further supported by our RNAseq analyses of LECs from WT and KO mice. They unambiguously demonstrate that the LECs of the KO mice have higher expression of pro-inflammatory genes (*Ilb, Ifi202b, S100A9, Csf3, Madcam1*) than their WT controls. Moreover, upon inflammation Clever-1 KO LECs up-regulated genes regulating migration, maturation, activation and tolerance (*Ackr2*, *Csf2, Tlr3*) whereas they downregulated several MHC I and II genes. *Ackr2* exerts its effects as a decoy receptor for quite a variety of chemokines regulating leukocyte trafficking, *Csf2* is important in DC homeostasis and activation and *Tlr3* is involved in tolerance (36–39). The downregulation of antigen-presenting molecules on the inflamed-LECs could possibly prevent the deletion of reactive T cells since they no longer would effectively present antigen improving DC-mediated T cell activation.

Based on these results, we like to consider that while Clever-1 deficiency at steady-state increases the pro-inflammatory state, additional inflammatory insults lead to tissue resolution to sustain homeostasis and prevention of overt inflammatory reactions. Whether this is a compensatory mechanism created during the lifetime of Clever-1 KO mice remains unknown. We have earlier shown that Clever-1 can directly bind B cells and CD8 T cells (12), although the counter-receptor on those cells has not yet been identified. The current RNAseq results demonstrate that lack of Clever-1 causes marked changes to various genes encoding important regulators and therefore, its absence may indirectly affect a multitude of immune functions in the body. The exact mechanisms remain to be elucidated.

However, since Clever-1 has been shown to regulate the immunosuppressive activities of human monocytes (40) and mouse macrophages (30) our results suggest that lymphatic Clever-1 has similar properties besides its involvement in the trafficking of DCs. As Clever-1 is currently a therapeutic target in cancer trials, the treatment is expected to have consequences also on lymphatics. Although KO mice are not equivalent to patients receiving anti-Clever-1 antibody, based on the findings of this work we envision that the lymphatics contribute together with macrophages to the immune activation seen in the trial patients (41).

## Data Availability Statement

RNA sequencing data have been deposited in the Gene Expression Omnibus database under the accession no. GSE148730 and the unique reagents and mice are available at request from the corresponding author.

## Ethics Statement

The studies involving human participants were reviewed and approved by the ethical board of Turku University Hospital. The patients/participants provided their written informed consent to participate in this study. All animal experiments were approved by The Finnish Act on Animal Experimentation (62/2006; animal license number 5762/04.10.07/2017).

## Author Contributions

ST, JD, DE, and RV performed the experiments. AT and KE performed the RNA seq analyses. MH and SJ designed and supervised the work. SJ wrote the first draft. All authors contributed to the article and approved the submitted version.

## Funding

This project was supported by the Finnish Academy, Sigrid Juselius Foundation, Jane and Aatos Erkko Foundation, the Finnish Cancer Foundation, Alfred Kordelin Foundation, and Finnish Cultural Foundation.

## Conflict of Interest

MH and SJ own stocks of Faron Pharmaceuticals.

The remaining authors declare that the research was conducted in the absence of any commercial or financial relationships that could be construed as a potential conflict of interest.
