# Supplementary material for: Lymphatic Endothelial Cell Activation and Dendritic Cell Transmigration Is Modified by Genetic Deletion of Clever-1
Source: Front Immunol. 2021 Mar 4;12:602122. doi: 10.3389/fimmu.2021.602122 (PMC7970002; doi:10.3389/fimmu.2021.602122)
Supplement: Supplementary file 1 [file DataSheet_1.docx]

**Supplementary Figure Legends**

**Supplementary Figure 1. Morphological appearance of lymphatics and the number of DCs in KO mouse skin are normal. (A)** Confocal imaging of dLNs of KO mice and their WT controls at steady state. **(B)** Confocal imaging of ear section of KO mice and their WT controls at steady state. Blue arrowheads indicate a collecting lymphatic vessel and red arrowheads indicate a capillary lymphatic vessel. **(C, D)** Confocal z-stack images of whole-mount ear dermis, indicating the normal structure of LVs of KO ear dermis compared to their WT control **(C),** and quantification of LYVE-1+ area **(D**); each dot represents one ear**. (E)** Flow cytometric analysis of ear-skin DCs at steady state; each dot represents one mouse. **(F)** quantification of the number of CD45+ cells in the ears skin of WT and KO mice. Each dot represents one mouse. **(G)** CD40 expression by DCs recovered from the draining popliteal LNs 18 hours after OVA (in incomplete Freund’s adjuvant) injection and analysed by flow cytometry; each dot represents 2 pooled animals. **(H)** Clever-1 expression in the ear skin 2 days after OXA challenge and comparison to KO mouse-ear staining at steady state. The arrowheads point to a lymphatic vessel. Data are expressed as means ± SEM. Two-tailed Student’s t-test. Scale bars **(A)** 200 µm, **(B)** 20 µm and **(C)** 200 µm and **(H)** 10μm.

**Supplementary Figure 2. Canonical pathway analysis of up-and down-regulated genes in WT and KO LECs upon footpad injection with OVA. (A)** Transcripts Per Million (TPM) of genes encoding LEC markers in Clever-1 KO mice and their WT controls at steady state; each dot represents two mice, Student’s t-test. **(B)** Transcripts Per Million (TPM) of genes encoding LEC markers in Clever-1 KO mice and their WT controls, one day after OVA (in incomplete Freund’s adjuvant) injection; each dot represents two mice, Student’s t-test. **(C)** Canonical pathway analysis of up-and down-regulated genes in WT and KO LECs upon footpad injection with OVA. *P < 0.05, **P < 0.01, ***P < 0.001 and ****P < 0.0001, Pearson’s correlation coefficient. **(D)** Genes in WT (upper panel) and KO (lower panel) LECs (Fold Change ≥ 2) belonging to the altered pathways shown in (Fig. 5G).

**Supplementary Figure 3. Different immune status in the skin of WT and KO mice.** Expression of cytokines in the ear skin of Clever-1 WT and KO mice at steady state, 24h-OXA treated ears (application of OXA onto the ears 24h earlier), 1 and 2 days after OXA challenge. Each data point represents one mouse. Data are expressed as mean ± SEM. *P < 0.05, **P < 0.01, ***P < 0.001 and ****P < 0.0001, One-way ANOVA with Tukey’s multiple comparison test.

**Supplementary Figure 4. Silencing Clever-1 in HDLECs alters their functional behaviour by increasing DC activation and T cell proliferation. (A)** Representative flow cytometry histogram of Clever-1 expression by LECs three days after siRNA silencing. Clever-1 was detected by AF647 conjugated 9-11 antibody. An isotype-matched antibody was used as a negative control. **(B)** Representative flow cytometry histograms and quantifications of MHCII, CD40 and CD80 expressed by moDCs co-cultured with siClever-1 or control-treated HDLECs for two days. An isotype-matched antibody was used as a negative control. (n=7, data are expressed as Boxplots where Min and Max values are shown as whiskers, Wilcoxon matched-pairs signed-rank test, *P < 0.05). **(C)** Flow cytometry plots of proliferating T cells and quantification of the percentage of proliferated T cells, co-cultured with moDCs and siClever-1 or control-treated HDLECs for 7 days. Each dot represents a different mixed leukocyte reaction. Data are expressed as mean ± SEM. *P < 0.05, two-tailed Student’s t-test.
