# Supplementary figures and images for: Lymphatic Endothelial Cell Activation and Dendritic Cell Transmigration Is Modified by Genetic Deletion of Clever-1
Source: Front Immunol. 2021 Mar 4;12:602122. doi: 10.3389/fimmu.2021.602122 (PMC7970002; doi:10.3389/fimmu.2021.602122)

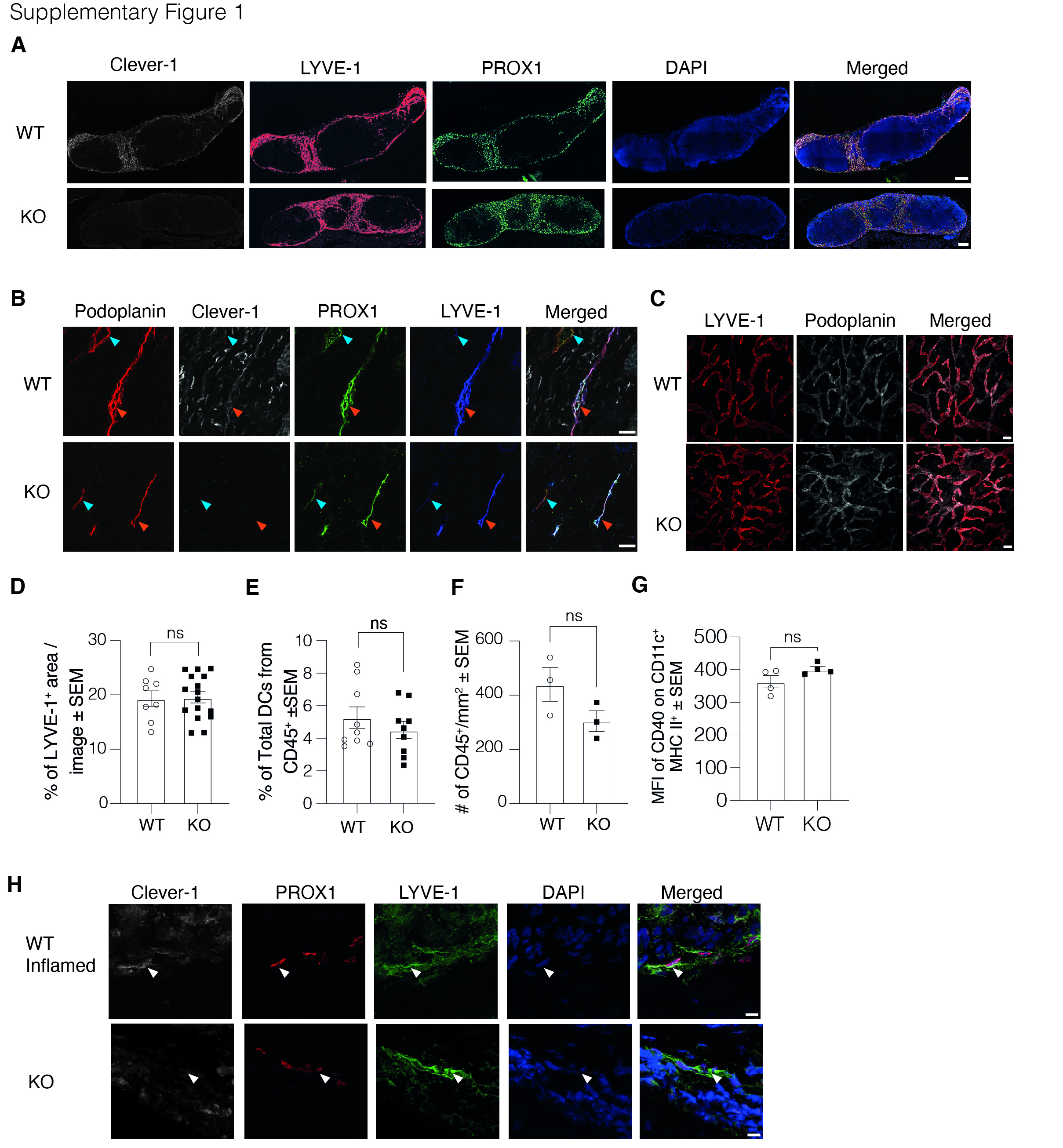

Supplement: Supplementary file 2 [file Image_1.jpeg]

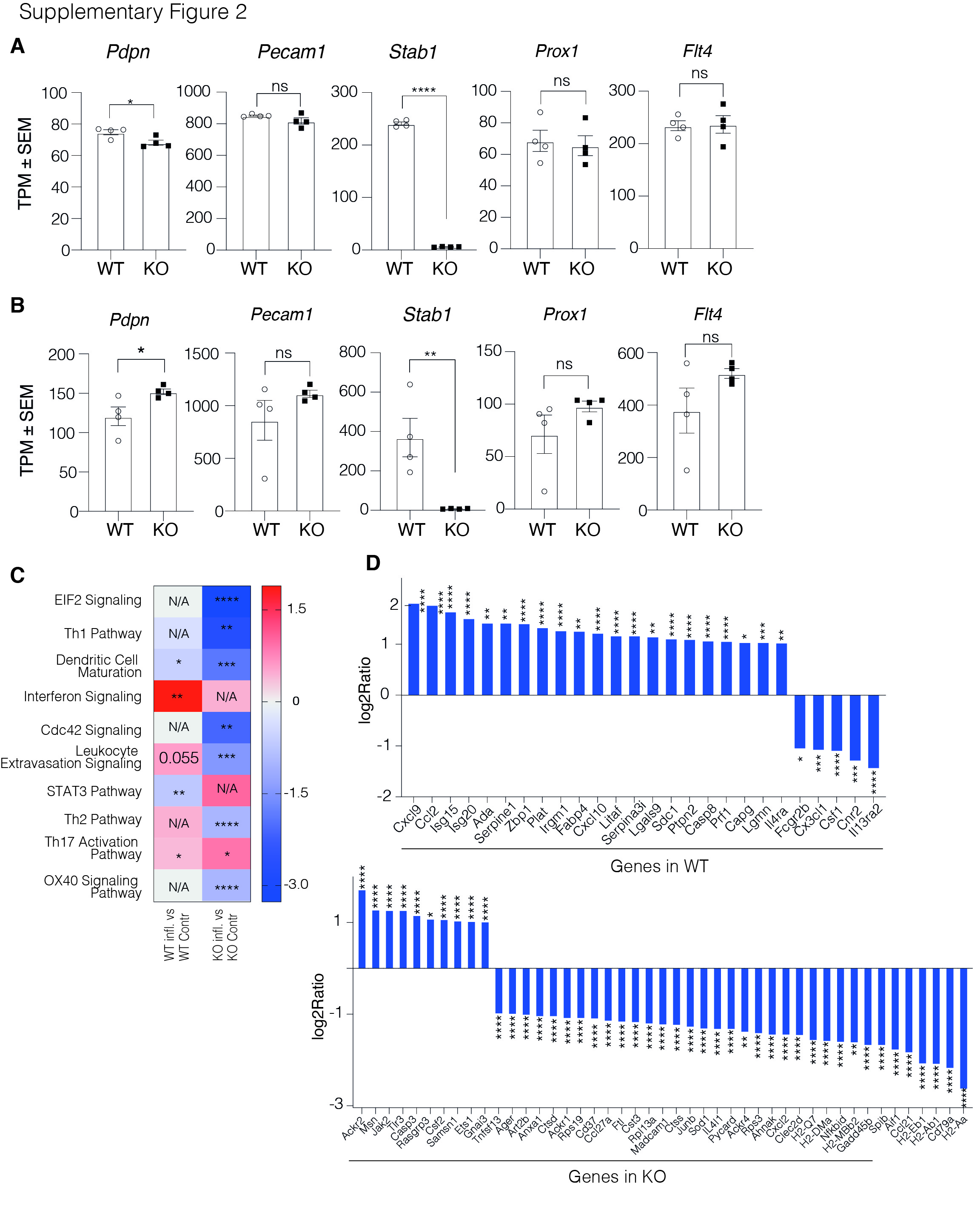

Supplement: Supplementary file 3 [file Image_2.jpeg]

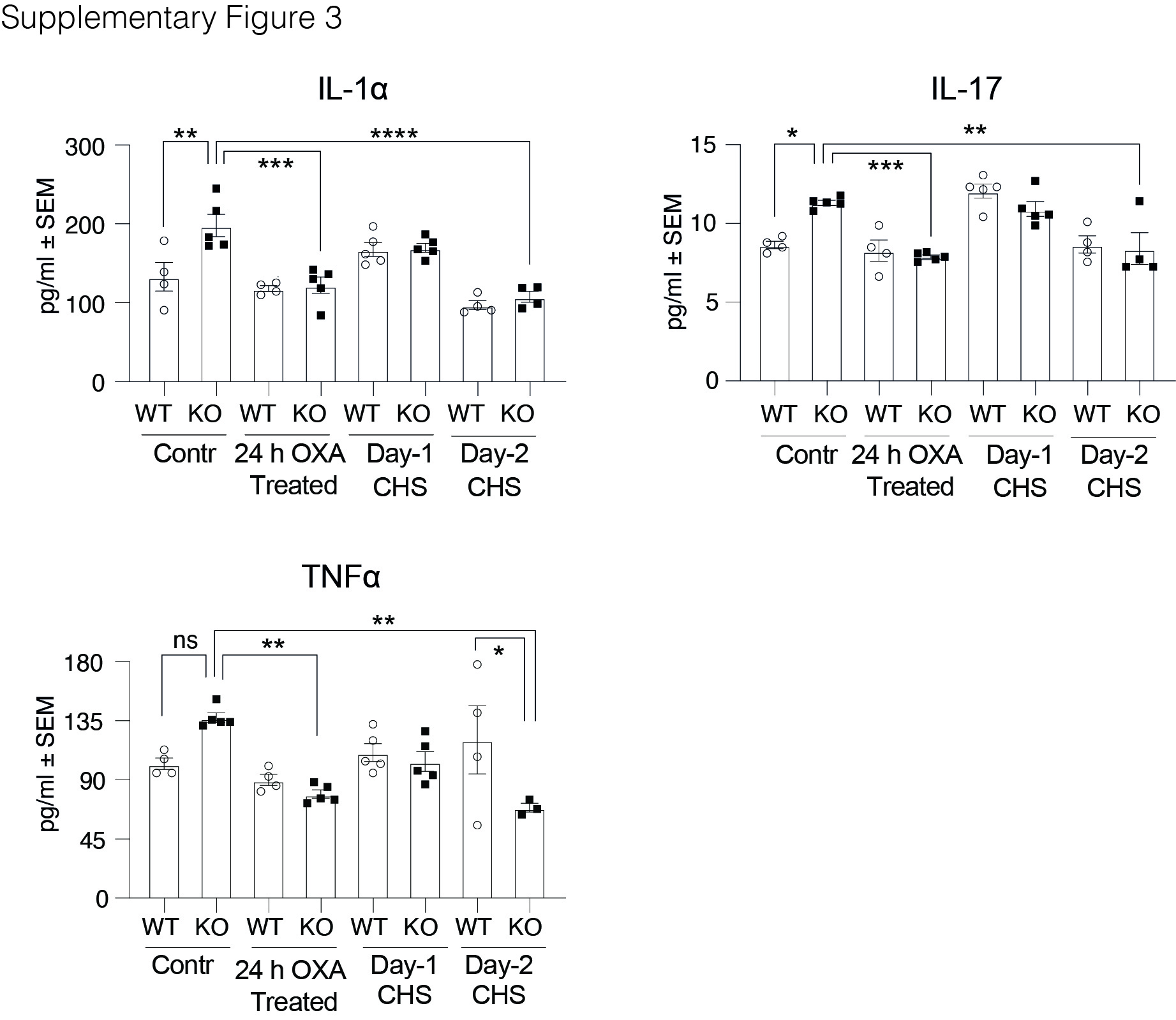

Supplement: Supplementary file 4 [file Image_3.jpeg]

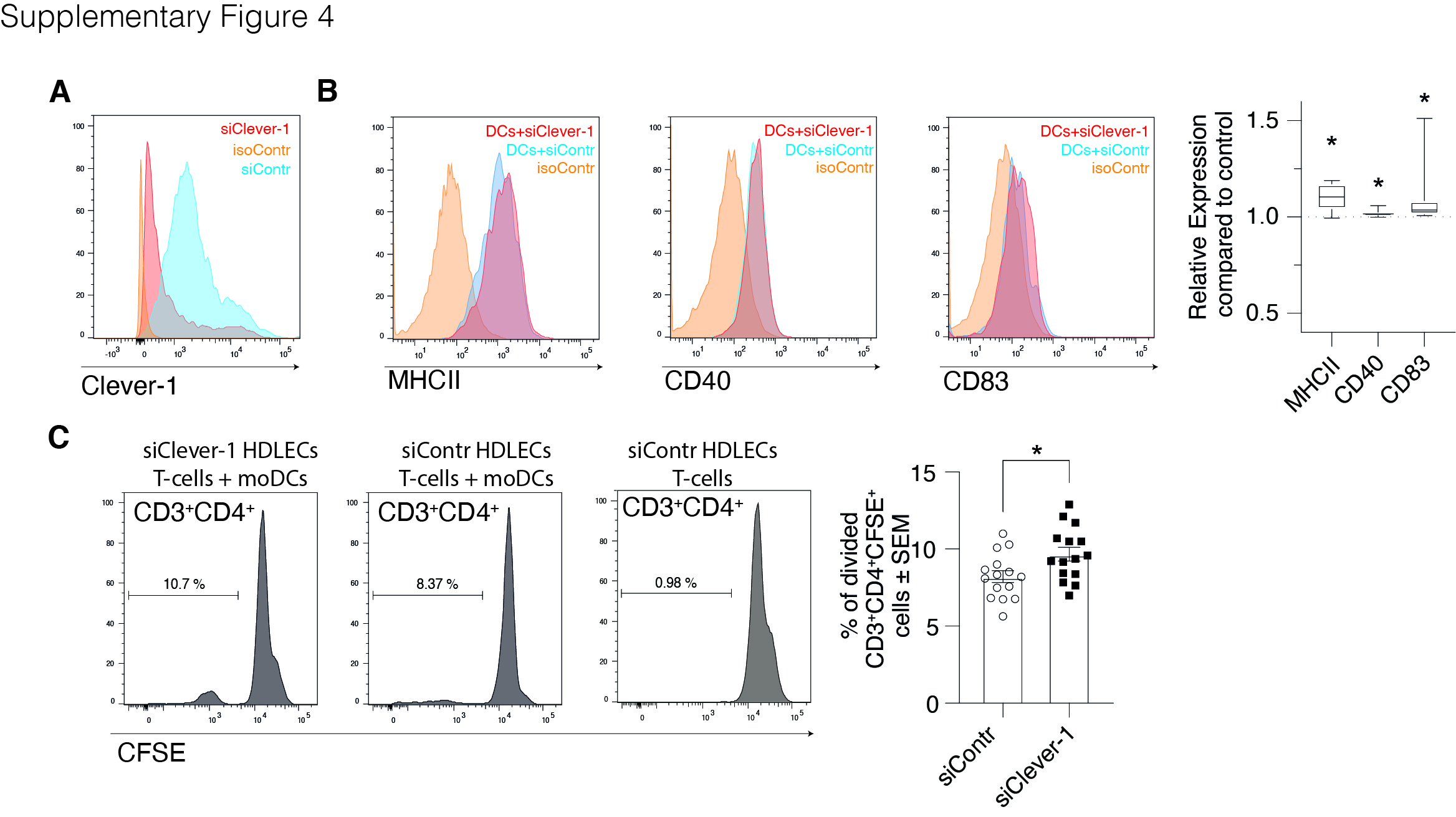

Supplement: Supplementary file 5 [file Image_4.jpeg]
